# Eradication of HIV and Cure of AIDS, Now and How?

**DOI:** 10.3389/fimmu.2013.00337

**Published:** 2013-10-18

**Authors:** Jielin Zhang, Clyde Crumpacker

**Affiliations:** ^1^Department of Medicine, Beth Israel Deaconess Medical Center, Boston, MA, USA

**Keywords:** HIV, memory CD4 T-cells, P21^Cip1^, epigenetics, hematopoietic stem cells

## Abstract

Recent studies have highlighted the importance of eradication of human immunodeficiency virus (HIV) and cure of acquired immunodeficiency syndrome (AIDS). However, a pivotal point that the patient immunity controls HIV reactivation after highly active anti-retroviral therapy [HAART or combination anti-retroviral therapy (cART)] remains less well addressed. In spite of the fact that both innate and adaptive immunities are indispensable and numerous cells participate in the anti-HIV immunity, memory CD4 T-cells are indisputably the key cells organizing all immune actions against HIV while being the targets of HIV. Here we present a view and multidisciplinary approaches to HIV/AIDS eradication and cure. We aim at memory CD4 T-cells, utilizing the stem cell properties of these cells to reprogram an anti-HIV memory repertoire to eliminate the viral reservoir, toward achieving an AIDS-free world.

## Introduction

Nothing stops an idea whose time has come. As in other scientific discoveries, the time has come for human immunodeficiency virus (HIV)/acquired immunodeficiency syndrome (AIDS) eradication and cure, emerging from three decades of cutting edge research on the HIV lifecycle, immune cell biology, and AIDS pathogenesis. Additionally, a burgeoning study of stem cells has broadened our knowledge of hematopoietic stem and progenitor cells (HSPCs) as well as memory CD4 T-cells that have properties of stem cells, to elucidate their decisive roles in the battle of modern medicine against HIV infection. We specifically address studies and approaches that focus on functional CD4 T-cell helped immune memory, a foundation and the mechanism of patient anti-HIV immunity, vaccination, a cure and a functional cure of AIDS after HAART.

## A Trinity of HIV/AIDS Eradication and Cure

A trinity of strategies on HIV/AIDS eradication and cure stems from the three theories or three scientific achievements: (1) defining CD4 T-cells in HIV/AIDS pathogenesis and immunity, (2) developing highly active anti-retroviral therapy to stop HIV replication; and (3) studying of ancient human endogenous retroviruses (HERVs) in the human genome, of which HIV is a modern example.

First, as in other viral infections, CD4 T-cells, specifically memory CD4 T-cells, govern the antiviral immunity against HIV infection. Whereas they commit themselves to antiviral immunity, CD4 T-cells, specifically memory CD4 T-cells, are the target cells of HIV. It is the dual role of memory CD4 T-cells in HIV infection that lays down the unique challenge for HIV/AIDS eradication and cure. Secondly, HAART blocks HIV replication in patients, controls viremia and viral transmission to people, which opens avenues to reconstitute patient HIV-specific immunity. However, no HAART so far has developed to stop the HIV provirus function. Finally, the research on HERVs shows that these viruses are the close relatives of HIV, and are present in the human genome without harm for generations. Harnessing the power of host cells employing to silence foreign DNA in the genome ultimately leads to a control of HIV provirus activity and therefore control of the viral reservoir.

In short, due to the dual role in HIV infection and anti-HIV immunity, memory CD4 T-cells have a dual role in control of viral reservoir and reconstitution of anti-HIV immunity after HAART. More than 28 approved drugs have been developed to target five different viral proteins in the HIV lifecycle, plus one targeting cellular protein CCR5 but none targeting on provirus to stop the viral RNA transcription (Table 1A). Besides HAART, immune therapies have been developed to reconstitute anti-HIV immunity, in which eight drugs are used in clinical trials, including bio-molecules for gene therapy and vaccine treatment (Table 1B). Despite all the anti-retroviral and immune treatments, reconstitution of patient anti-HIV immunity and control of the HIV reservoir remain elusive. The mechanisms that the host cell employs to permanently silence HERVs are explored, but little is known about how to utilize these heritable epigenetic machineries to silence HIV provirus and control HIV reservoirs in patients (1–15).

**Table 1:** **Anti-retroviral and immunotherapeutic drugs used in HIV eradication and AIDS cure**. **(A)** More than 28 approved drugs target five different viral proteins in the HIV lifecycle, plus one targeting the cellular protein CCR5 but none targeting at provirus transcription. **(B)** Immunotherapies are developed in addition to HAART to reconstitute anti-HIV immunity, in which eight drugs are being used in clinical trials, including the bio-molecules for gene and vaccine therapies. Although a decreased viral load and an increased CD4 T-cell number are reported specifically after vaccine treatment in patients with or without cART, the results of immunotherapy approaches remain in phase I or II, and to be confirmed by phase III trials for their efficacy, side effects and safety.

A HAART block multiple steps in HIV lifecycle		B Immune-based therapies
Binding and Fusion	Maraviroc, Fuzeon …	Aralen (Chloroquine phosphate)
Reverse Transcription	Emtriva, Edurant …	DermaVir (therapeutic vaccine)
Integration Transcription	Raltegravir, Dolutegravir …	Interleukin-7 (IL-7)
		Lexgenleucel-T (VRX-496; gene therapy)
Assembly	Aptivus, Ritonavir …	Plaquenil (hydroxychloroquine)
Budding	Bevirimat	Proleukin (aldesleukin, Interleukin-2, or IL-2)
		SB-728-T (gene therapy)
		Vacc-4x, FIT-06 … (therapeutic vaccine)

## The Role of Memory CD4 T-Cells

The key player in AIDS pathogenesis and cure is the memory CD4 T-cells. These cells are predominantly quiescent but are capable of intermittent self-renewal and long-term survival, meaning that these cells have stem cell like properties of HSPCs at the cell-cycle control, self-renewal, asymmetric division, and differentiation into effector cells (16–32). Let us briefly view the mechanisms, which memory CD4 T-cells employ to play the pivotal roles in eradication of HIV and cure of AIDS.

**Figure 1:**
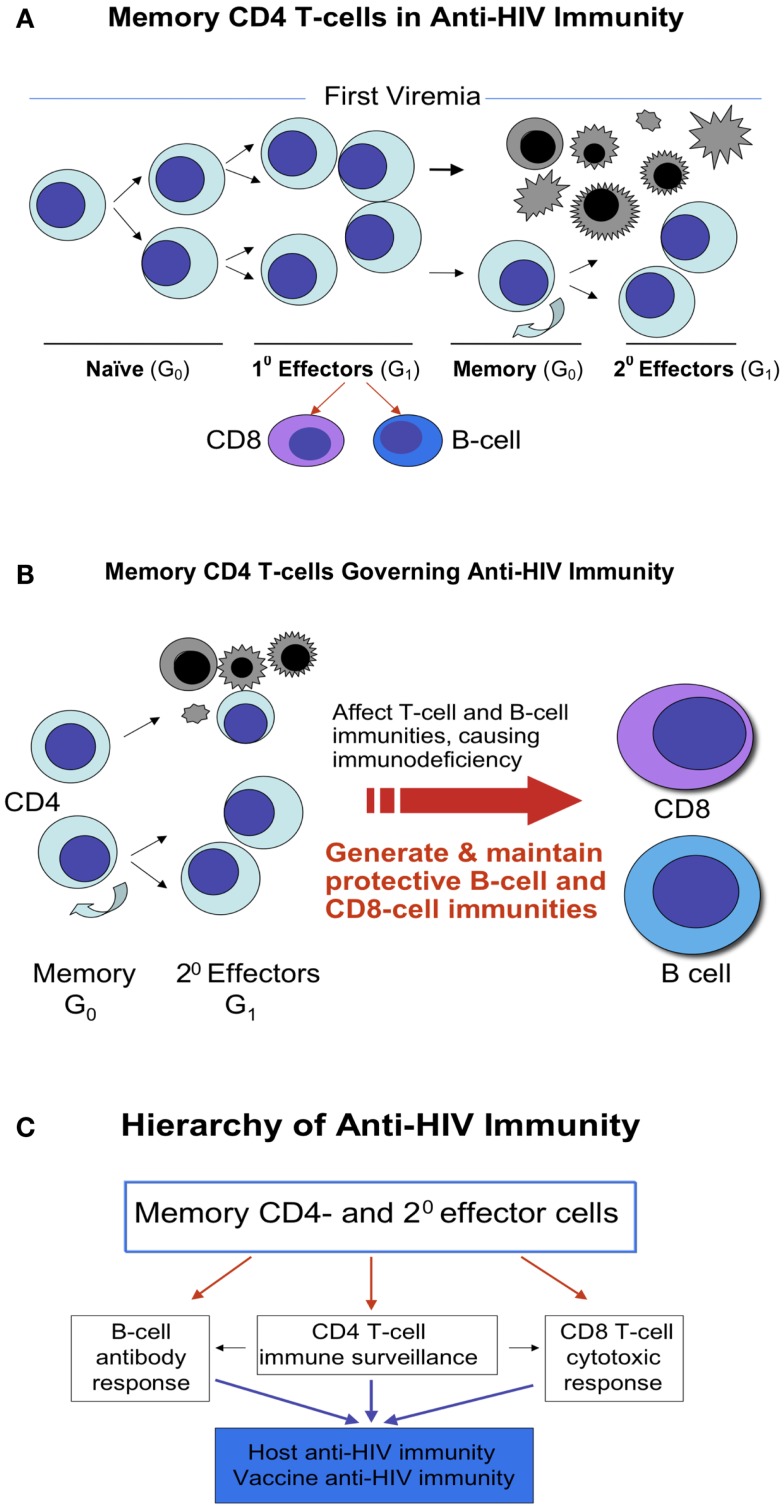
**Memory CD4 T-cells in anti-HIV immunity**. **(A)** Naïve CD4 T-cells are derived from CD4 lineage progenitors. Upon meeting a pathogen, such as HIV, naïve CD4 T-cells differentiate into antigen specific effector cells. Some of them die due to the viral infection or their biological destinies, whereas some of them survive and become memory cells and further develop into secondary effector cells. These memory cells retain the stem cell properties of asymmetric division, self-renewal, and differentiation into effector cells. **(B)** When encountering HIV again, these antigen specific memory T-cells, in this case HIV specific, repeat the process in **(A)**, and majority of effector cells die but some of memory cells sustain to continue the cycle. In general, the earlier the application of HAART is, the more memory cells preserve or survive. **(C)** The memory CD4 T-cells act as a commander in chief, in the leadership position for launching anti-HIV immunity as these cells govern functions of both CD8-cells and B-cells, regulate both cellular and humoral immunities, by which determine the prognosis of AIDS.

Upon naïve CD4 T-cells meeting a pathogen, in this case, the HIV, these cells differentiate into antigen specific effector cells. Some of them die due to the viral infection or their biological destinies, but some survive and become memory cells and further develop into secondary effector cells. When encountering HIV again, these antigen specific T-memory cells, in this case HIV specific, replicate and differentiate into the secondary effector cells (Figures 1A–C). The HIV specific effector cells, specifically memory CD4 T-cells, play decisive roles not only in anti-HIV immunity (33–35) but also in AIDS pathogenesis, since these cells govern functions of both CD8-cells and B-cells, regulate both cellular and humoral immunities, thereby determine the prognosis of AIDS (Figure 1B).

Memory CD4 T-cells are heterogenous and proposed to exist in at least three classes dubbed central memory (T_CM_), transitional memory (T_TM_), and effector memory (T_EM_) T-cells, plus previously identified Th1 and Th2 memory cells and recently defined Th17 memory cells (16–32). Among them, secondary effector cells of Th17 are the key players in mucosal immunity yet these cells are unable to completely recover from the early HIV destruction even after successful HAART (36, 37).

Likewise, there are memory CD8 T-cells. Differing from memory CD4 T-cells, however, generation of functional CD8 T-cell memory requires the help of CD4 T-cells. A defective CD8 T-cell memory following acute infection without CD4 T-cell help has been identified in numerous animal model studies. Many efforts are complied to rescue the CD4-unhelped CD8 T-cell memory to restore the viral specific immunity (38–45). Scarce studies, however, have been conducted on CD4-unhelped CD8 T-cell memory in HIV patients, who have an inverted ratio of CD4/CD8 and a signature defective CD4-unhelped CD8 T-cell memory following the HIV acute infection (46–53). Considering that vaccine strategies are built on the immune cell memory function and CD8 T-cell memory needs the help of CD4 T-cells, elucidation of how CD4 T-cells program CD8 memory or how CD4 T-cell is required for the long-term maintenance of CD8 memory T-cell numbers and functions will add great value to reconstitution of patient anti-HIV immunity (38–53). Moreover, recent studies show that CD4 T-cells control the proliferation, accumulation, and activation of natural killer (NK) cells (54, 55). HIV induced loss of CD4 T-cells plus an impaired CD4 T-cell activity appear to contribute to progression of liver fibrosis in patients co-infected with HIV and hepatitis C virus (HCV) (56). In light of these results, no matter what mechanism of CD8 T-cells or NK cells employ to kill HIV, it is clear that memory CD4 T-cells act as a commander in chief in launching and orchestrating the immunity against HIV (Figure 1C). Without this leadership, small local battles may be won, but the whole war is lost.

We and others have focused on studying the repertoire function of memory CD4 T-cells, that is the function of a group of memory CD4 T-cells governing the anti-HIV immunity. We examined the patterns and the courses of surviving memory CD4 T-cells in which they develop into effector cells and their function with memory CD8 T-cells after the viremia. We believe that the repertoires of memory CD4 T-cells after the HIV attack decide the anti-HIV immunity and the status of HIV/AIDS pathogenesis in individuals (16–32). If a close to normal repertoire of memory CD4 T-cells survive after the early viremia, meaning the first and second viremia (57–60), the HIV replication can be controlled by the host immunity with or without HAART. In most HIV infections, however, the repertoire of memory CD4 T-cells is damaged and destroyed. This not only results in HIV/AIDS pathogenesis, but also a big challenge in reconstitution of anti-HIV immunity. The loss of immune memory repertoires of CD4 T-cell also sets the biggest hurdle to the development of HIV/AIDS vaccine since a damaged CD4 T-cell memory and a CD4 T-cell unhelped CD8 T-cell memory can only lead to a defective vaccine and an aborted vaccination.

Memory CD4 T-cells have a pivotal role in HIV/AIDS eradication and cure. Not only are these cells the key cells in HIV infection and immunity, but these cells also have the stemness properties of HSPCs. The asymmetric dividing of memory CD4 T-cells provides the long-living memory cells serving as a major source of HIV reservoir on the one hand, and on the other hand, provides the short living effector cells serving as the targets of HIV replication. The stemness properties of memory CD4 T-cells stem from HSPCs. What is the role of HSPCs in HIV/AIDS eradication and cure? We and others have studied HIV infection of HSPCs. We have found that HSPCs are resistant to HIV infection, despite that other bone marrow cells, including blood lineage progenitor cells, are accessible to HIV infection (61–66). At the level of HIV/AIDS eradication and cure, however, the techniques and methodologies for studying of HSPCs should be utilized to study memory CD4 T-cells, peculiarly for translational research and multidisciplinary collaborative studies.

Of note, recent studies reveal that memory CD4 T-cells preferentially reside and rest in the bone marrow, and virus-specific memory CD4 T-cells are abundant in unexposed adults (67, 68). Accordingly, HIV patients are capable of rebuilding HIV specific immunity via these abundant memory CD4 T-cells after HAART, responding to HIV as a novel pathogen to take the second chance reestablishing anti-HIV immunity from an abundant *in vivo* pool of memory CD4 T-cells (16–32, 67, 68). Tackling the second chance to rebuild patient immunity against HIV after HAART is the prime goal to achieving an AIDS-free world.

## HERVs and HIV Provirus

A rationale for urging that HIV/AIDS can be eradicated and cured is further backed up by the third theory: the ancient HERVs are present in the human genome for many generations without harm. HERVs are the close relatives of HIV, which are kept silenced permanently in the human genome via cellular epigenetic machineries (1–11). The past and current studies have revealed that the epigenetic regulation is the prime mechanism which host cell employs to protect its genome integrity against the invasion of foreign DNA including HIV DNA (the provirus) (1–11, 15, 69–71).

Epigenetic regulation consists of DNA methylation at CpG dinucleotides, covalent modification of histone proteins in chromosomes, and interplay of non-coding RNAs (ncRNAs) in the genome. Among them, DNA methylation is the best-understood and most thoroughly studied epigenetic regulation, of which DNA methyltransferase 3b (DNMT3b) is the main enzyme for *de novo* DNA methylation. Epigenetic regulation is fast, potent, heritable, and it changes the gene expression at a transcriptional level without changing of DNA sequences, the very step in HIV lifecycle that HAART is off target (Table 1). While no HAART regimen to date silences HIV proviral gene transcription, DNA methylation is, however, a showcase in silencing gene expression of HERVs included HIV in the human genome and also in controlling of retroviral diseases in animal model studies (1–11, 15, 69–71). Note that with HAART, HIV is kept as non-virulent but remains in the reservoir, which consists mainly of memory CD4 T-cells (72–79). Epigenetic regulation is an evolutionarily developed mechanism with a signature of silencing foreign DNA expression, like blocking the transcription of HIV provirus, while maintaining cellular gene function. Considering the double role of memory CD4 T-cells in HIV immunity and infection, we believe utilizing epigenetic regulation to silence HIV in memory CD4 T-cells is a win–win strategy, which silences HIV without hurting cellular gene expression, and stops HIV provirus without losing lives of memory CD4 T-cells who intend to be long-memory, long-living, and everlasting to patient HIV-specific immunity.

It is widely accepted that when exposed to antigenic stimulus, the normal immune system mounts an appropriate response and then returns to relative quiescence after clearing the antigen. Memory CD4 T-cells govern this process by returning to a quiescent status (G_0_/G_1_), which allows the innate and adaptive immune systems to go back into a reset or surveillance state. HIV disrupts this balance by provoking chronic immune activation, cytokine elaboration, and ultimately alteration of the microenvironment of the immune system. HAART restores the quiescence by blocking HIV replication without silencing the provirus in host cells. One can promote an immune activation from the immune quiescence after HAART, or one can utilize the intrinsic cellular epigenetic machineries to preserve the quiescence induced by HAART for these memory CD4 T-cells. Considering the rapid progress in studying of immune cells and their niches, we can harness the intrinsic epigenetic machineries to silence provirus activity in the memory CD4 T-cells and sequentially promote the CD4 T-cell helped CD8 T-cell memory. We consider the epigenetically regulated memory CD4 T-cells and the CD4 T-cell helped CD8 T-cell function to be the foundation of an AIDS cure, either sterile or functional. Specifically, one can utilize DNA hypermethylation to permanently silence HIV provirus in memory CD4 T-cells akin to silencing HERVs, and such strategy is being studied and developed (1–11, 15, 69–71, 80).

## Cellular Factors in HIV/AIDS Eradication and Cure

Cellular factors TRIM5α, APOBEC3G, SAMHD1, etc. are studied by many investigators to define the effects of these proteins in HIV infection and AIDS cure. P21^Waf1Cip1Sdi1^ (p21) is one of them. P21 is a cyclin dependent kinase inhibitor regulating cell cycle, particularly in blood cells. We study p21 because p21 plays an indispensable role in immune cell quiescence, differentiation, senescence, and apoptosis, in addition to the role of this single cell checkpoint protein playing in HSPCs resistance to HIV infection (61–64, 81–90). Importantly, p21 dictates cell-cycle status of HSPCs whereas memory CD4 T-cells have the properties of HSPCs (16–32). Based on memory CD4 T-cells playing a dual role in reconstitution of anti-HIV immunity and control of HIV reservoir after HAART, revelation of the mechanism of p21 in cell-cycle control, turnover, senescence, and apoptosis of memory CD4 T-cells is pivotal for reprograming patient HIV-specific immunity and eliminating the viral reservoir.

Molecularly, p21 is activated by damage to cellular DNA, via p53 dependent and in-dependent pathways. After p21 activation, there are two possible outcomes for blood cells: being quiescent and arresting in G_1_ phase, or being senescent and going into apoptosis (81–90). The former, on the one hand, is the base of HIV reservoir, and the latter, on the other hand, is the platform to get rid of this reservoir. Therefore, p21 is imperatively a focus point not only in understanding of memory CD4 T-cell differentiation and renewal, but also in apoptosis and turnover, all directly affecting the dimension of HIV reservoir *in vivo* (61–68, 72–79, 81–94).

We have studied p21 in HIV infection since 2002 (22, 61–64). At the beginning, we investigated the function of p21 in HSPCs after HIV infection. Today, we propose to continue to examine the role of p21 in activation, senescence, and apoptosis of immune cells, in order to restore a memory CD4 T-cell repertoire to reconstitute, or in a more accurate term, to reprogram the CD4 T-cell helped CD8 T-cell and B-cell immune memories to rebuild patient HIV-specific immunity.

In our previous studies, we have demonstrated that p21 plays a central role in HSPCs resisting HIV infection. The p21 mediated resistance to HIV infection in blood stem and progenitor cells occurs at the viral integration step, by which makes HSPCs a sanctuary in resisting HIV provirus formation (61–63). Our recent studies further showed that p21 directly bound to HIV long terminal repeat (LTR) and integrase (IN), respectively (data not shown).

To summarize, our molecular studies show that p21 blocks HIV infection in HSPCs at the viral integration step. P21 directly interacted with LTR and IN, indicating that blood cells or the immune cells have an innate resistance against retroviral infection at the level of preventing viral DNA integration. Additionally, other investigators have found that p21 inhibits HIV infection in macrophages and CD4 T-cells, respectively, by different cellular mechanisms and pathways (91–94). From study of p21, we realize that as a principle of immunotherapy and vaccination, the induced immunity has to be efficient enough to target or to block a key step in the viral lifecycle. For a retrovirus like HIV, the key steps include: (1) to stop HIV integration so the provirus cannot be formed in the host genome, (2) to stop the transcription after the provirus formation so the viral DNA is kept silent until the cell senescence or apoptosis. So far, no drug in HAART targets the second step. P21, however, appears to have functions at both these steps. Moreover, p21 is a cellular factor having a tumor suppressor function, which makes p21 a good candidate to synergistically regulate the immunity with either chemotherapy or HAART in AIDS patients with or without tumors.

## Molecular and Cellular Strategies Tackling HIV Reservoir

Due to the stem cell like properties, not only are memory CD4 T-cells the key cells to reconstituting patient anti-HIV immunity, but also a major HIV reservoir. Genetic and epigenetic approaches are being developed to eliminate HIV reservoirs, including targeted genome cleavage by zinc finger nucleases (ZFNs) (12–14), and epigenetic modulations such as histone deacetylation, histone methylation, and DNA methylation (1–11, 15, 69–71). As previously stated, the difference between genetic and epigenetic approaches on eradicating HIV infection is that the former changes the host cell genome, the blue print, but the later does not. Epigenetic regulations control the gene expression without changing the host cell DNA sequences.

The targeted genome cleavage utilizing ZFNs has shown success in CCR5 knockout T-cells isolated from HIV patients to protect these lymphocytes from HIV infection. These approaches are now moving into the clinic with CCR5-specific ZFNs being used to disrupt CCR5 in autologous T-cells transplanted into HIV-infected patients (clinicaltrials.gov, NCT00842634, NCT01044654). As for targeting and cleaving regions within latent HIV genomes, however, ZFNs based approaches are still in development. The big challenge is the accessibility of ZFNs to HIV DNA, due to the formation of repressive chromatin at the HIV promoter, including loss of activating histone modifications, presence of repressive modifications, and presence of DNA methylation that has been implicated in HIV latency (1–11, 15, 69–71). The recognition and cleavage of ZFNs on HIV DNA may further be compromised by the presence of heterochromatin and possibly nucleosome positioning (15, 69–71). In sum, the efficacy of approaches like ZFNs in control of the viral reservoir depends on the epigenetic status of HIV DNA in the human genome.

Regarding the implementation of epigenetic approaches to eradicate the viral reservoir, the most widely discussed method is to using histone deactylase inhibitors such as suberoylanilide hydroxamic acid (SAHA) to reverse latency in resting CD4 T-cells with the assumption that the cells will then die from viral cytopathic effects or be lysed by host cytolytic T-cells after disruption of the latency (95–99). Current findings, however, show that the reactivation or latency disruption might not be sufficient to eradicate the HIV although some of these inhibitors are advanced to clinical trials (95–99).

Strategies like stem cell transplantation, therapeutic vaccination, and increasing the concentrations anti-retroviral compounds in the sanctuary sites of the viral reservoir have been applied in eradication of HIV reservoirs. Particularly, multiple means to increase drug concentrations in sanctuary sites are being investigated, for improving the efficacy of HAART, including modification of currently utilized drugs, blockade of transporters and enzymes that affect drug metabolism and pharmacokinetics, and the local drug administration (100, 101).

Despite all above approaches, the inability of the immune system to recognize cells harboring latent virus and inability to eliminate cells actively producing virus remain the biggest challenge toward HIV/AIDS eradication and cure. Investigators look at new strategies to unravel the virus-host interactions that lead to viral persistent infection and memory CD4 T-cell quiescence, and discuss the rationale for combination of novel treatment strategies with available anti-retroviral treatment options to cure HIV. One of the strategies, which is used in cancer immunotherapy to kill cancer cells by bio-engineered mesenchymal stem cells (MSC), is in development and can apply to eliminate quiescent memory CD4 T-cells harboring HIV. This strategy aims to attack viral reservoirs throughout the body, including the central nervous system, gut-associated lymphoid tissue, lymph nodes, and tissue macrophages, since MSC reside not only in bone marrow but also in other tissues, and differentiate into multiple stromal cells throughout the human body. MSC periodically circulate in blood, tend to lodge in lymphatic tissues and further divide into progeny cells (102–104). Bio-engineered MSC can approach the compartments that HAART cannot reach, not only eliminating viral reservoir but also restoring a niche that replenishes HIV-specific memory CD4 T-cells and executes the HIV-specific memory, function, and immunity.

**Figure 2:**
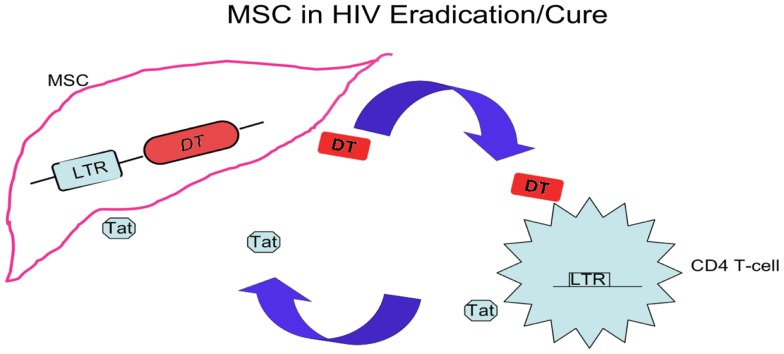
**A stem cell based treatment**. In addition to hematopoietic stem cell transplant, other stem cells, such as MSC, can be used in HIV/AIDS eradication and cure via relatively easy cellular source and process. The modified MSC aim at compartments refractory to HAART, and this strategy is derived from the cell-based therapy killing cancer cells by pro-drug delivery with MSC. After transduction of LTR-DT gene and interaction with cells infected by HIV expressing Tat, these MSC are activated and diphtheria toxins (DT) are generated. The toxin kills the infected CD4 T-cells and nearby cells included MSC producing the toxin, by which eliminates the HIV-infected cells and the HIV reservoir.

As shown in Figure 2, MSC bearing an LTR-diphtheria toxin (DT) gene can be activated after exposure to HIV Tat, and express DT to kill the cells expressing Tat (105). The MSC bearing LTR-DT act as a time bomb, to die and explode, killing cells of active HIV infection with Tat expression. These MSC are not only timed and directed, but also outnumbered their enemies, waiting patiently to kill the enemy cells with a potency not regarding their own survival. Such stem cell based therapy reinforces HIV-specific immune responses by nourishing memory CD4 T-cells while eliminating cells with active HIV infection, helping to reach the goal of eradication of viral reservoirs or the alternative aim of a functional cure for HIV.

**Figure 3:**
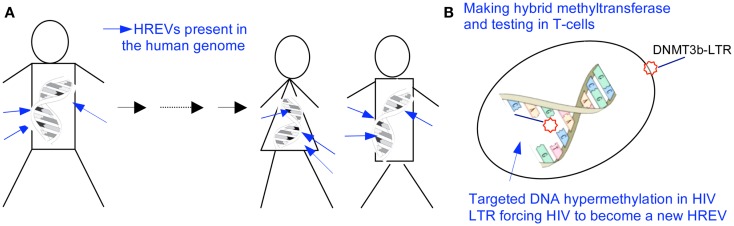
**Silencing HIV reservoir via epigenetic machineries**. **(A)** Methylated HERVs are passed for generations without causing retroviral diseases, and a targeted hypermethylation at the DNA sequence of HERVs has been recognized to be the mechanism silencing gene expression of ancient endogenous retroviruses in the human genome (1–11). **(B)** HIV is a modern example of HERVs. A targeted hypermethylation at the HIV promoter will be possible to genetically and sterilely block HIV RNA expression, mimicking what occurs to HERVs. DNMT3b is the prime enzyme catalyzing the transfer of methyl groups to cytosines of a targeted gene to silence the gene expression in the human genome, specifically to endogenous retroviruses and transposons. LTR contains the most conserved DNA sequence in a latent HIV. DNMT3b-LTR binds to the proviral LTR by base pair affinity, in a time of either RNA transcription or DNA semi-conservative replication. The bio-molecule binding to the HIV LTR leads to a heritable, stable DNA hypermethylation at the HIV promoter (1–11, 15, 69–71, 80).

Furthermore, in contrast to activating quiescent HIV and cells by histone deactylase inhibitors like SAHA, another innovative strategy is developed to permanently silence but not to activate the integrated HIV and prevent the spread of infection, in line with the results in studying of HERVs (Figure 3). One of these studies reports that a chimerical protein IN3b, consisted of a HIV-1 IN domain recognizing the HIV LTR and the catalytic domain of DNMT3b, decreases LTR-associated HIV genome expression and HIV replication, to cause the long-term silencing of HIV replication by permanently silencing the HIV promoter in the cell genome (80).

Human immunodeficiency virus is akin to HERVs that are methylated in the human genome and passed for generations without causing retroviral diseases (1–11, 15, 69–71). A hypermethylation at the DNA sequence of HERVs and silencing of viral gene expression have been recognized as the underpinning mechanisms (1–11, 15, 69–71). Thus, a targeted hypermethylation at the HIV promoter leads to and results in a sterile blocking of HIV RNA expression, mimicking what occurs to HERVs. Biologically, an initial DNA methylation is followed by a histone modification resulting in the secondary epigenetic modifications at the chromosomal level. The primary and secondary modifications will permanently lock the HIV promoter, forcing the HIV to become another endogenous retrovirus in the human genome. This process significantly differs from an assumed HIV latency that occurs at a histone level, responding to numerous stimuli and not being stable or heritable (1–11, 15, 69–71).

**Figure 4:**
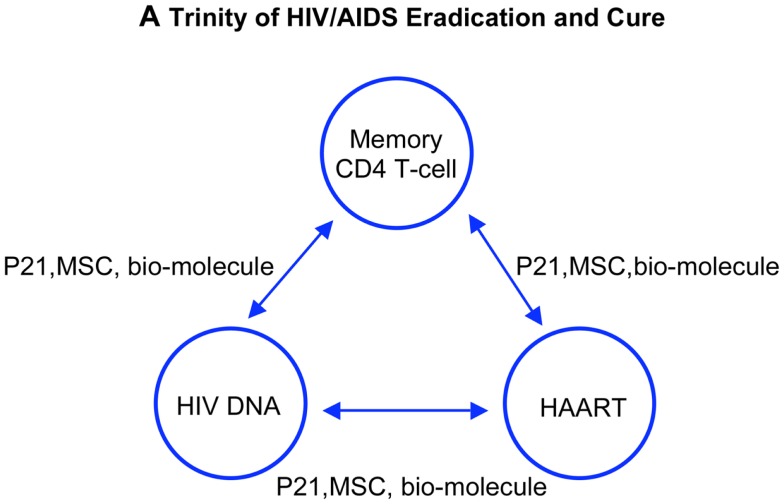
**A trinity of HIV eradication and AIDS cure**. Strategies and approaches stem from the three achievements in the field of studying HIV/AIDS. Each alone, however, cannot achieve the goal toward an AIDS-free world. HAART decreases the HIV DNA thus increases the memory CD4 T-cell function. Increased memory T-cell function in turn controls the activity of viral DNA therefore increases the efficacy of HAART. Approaches with impacts on the triad, such as modulating memory CD4 T-cell turnover, eliminating HIV reservoir, and silencing HIV DNA, ultimately lead to reprogram memory CD4 T-cell repertoire and patient anti-HIV immunity. An AIDS-free world will be built on patient immunity against HIV, regulated by memory CD4 T-cells endowed with long living, self-renewing, and differentiating into antigen specific effector cells to control HIV provirus activation.

To sum up, although HAART effectively controls the viremia and viral transmission, the patient HIV-specific immunity and the viral reservoir remain as two big hurdles in HIV/AIDS eradication and cure. For an AIDS cure or a functional cure of which host and virus co-exist for a long term in the absence of toxic and costly drugs, the patient HIV-specific immunity after HAART is indisputably a key (46, 106, 107). This is a matter of fundamental principle in modern medicine regarding the roles of human immunity vs. chemical compounds in a human body: who has the final say on eradication of HIV and cure of AIDS? Can HAART replace patient immunity to cure AIDS? Can we develop an HIV vaccine without immune cell memory? Finally, what have we learned from cancer therapy, and is there a connection in lack of antigen specific immune memory between AIDS and cancer patients after the respective chemotherapies? Others have provided answers and approaches toward the HIV/AIDS eradication and cure, and we add more to this as follows (Figure 4).

## Concluding Remarks

Retrospectively, infectious diseases have been cured for only one reason – human immunity controls the replication of, and/or gets rid of the pathogen, with or without anti-microbial drugs. In spite of the help of anti-microbial compounds, it is the human immune system that has the final say on a disease being cured or not. Both innate and adaptive immunities are indispensable, and numerous immune cells take part in and each plays a pivotal role, but memory CD4 T-cells have an undisputable role as the commander in chief to organize all immune actions against an invading pathogen (16–32, 56, 38–55, 57–60). The function of memory CD4 T-cells is, undoubtedly, the foundation of both adaptive immunity and its branch applied to medical science, the vaccine and vaccination.

Being in an era of modern medicine after the human genome project, in the center of burgeoning study on stem cells and translational therapies, after three decades of intensive research on the lifecycle of HIV and pathology of AIDS, with a team effort of investigators in multiple disciplines and cross-fields, similar to others, we present our opinions and approaches toward achieving an AIDS-free world that are derived from our two decades of studying HIV replication, immune cell biology, and AIDS pathology. We have reviewed how we will work from a trinity of strategies to eradicate HIV and cure AIDS, to reprogram the immune repertoire function of memory CD4 T-cells and reconstitute patient anti-HIV immunity to cure AIDS with or without HAART. Generation of effective anti-HIV vaccines has been a particularly difficult task. Therefore, attempts to cure AIDS via early administration of anti-retroviral therapies maybe in the long run a more successful and rewarding strategy. To conclude, utilizing powers of HAART, either by developing compounds or bio-molecules to stop provirus transcription or reprograming memory CD4 T-cells to reconstitute patient HIV-specific immunity, we can pave a road for generating effective AIDS vaccines; by deciphering the mechanisms of memory CD4 T-cell guided immunity, we may open a door reconstituting patient immunity against not only cancer but AIDS.

## Authors Contribution

Jielin Zhang wrote and Clyde Crumpacker edited the paper. We greatly appreciate the valuable comments of Dr. Joseph Sodroski on this paper.

## Conflict of Interest Statement

The authors declare that the research was conducted in the absence of any commercial or financial relationships that could be construed as a potential conflict of interest. This review is bestowed on Boston Strong for lost and injured lives, and for humanity against terror, disability, and diseases.
